# Editorial: Rediscovering Local Landraces: Shaping Horticulture for the Future

**DOI:** 10.3389/fpls.2019.00126

**Published:** 2019-02-12

**Authors:** Spyridon A. Petropoulos, Lillian Barros, Isabel C. F. R. Ferreira

**Affiliations:** ^1^Laboratory of Vegetable Production, Department of Agriculture, Crop Production and Rural Environment, University of Thessaly, Volos, Greece; ^2^Centro de Investigação de Montanha, Instituto Politécnico de Bragança, Bragança, Portugal

**Keywords:** biodiversity, common bean, fruit quality, horticulture, genetic resources, legumes, lettuce landraces, tomato landraces

Local landraces constitute a valuable genetic pool of increased diversity that can be exploited in breeding programs for the production of new commercial cultivars with targeted traits. Despite the acknowledged importance of local landraces, recent market trends and modern horticultural production systems have shifted farmers and related sectors to cultivation of commercial cultivars and hybrids due to their high yield and uniformity of the final product. However, this trend results only to short-term benefits while it puts in danger the valuable genetic resources of local horticultural landraces which may be proven useful under a global climate change. Moreover, consumers' concerns about food quality, its origin and production process as well as geographical indication (GI) of products are in increasing demand. Especially for the EU, Protected Designation of Origin (POG), Protected Geographical Indication (PGI) and Traditional Specialties Guaranteed (TSG) labels have been established, while according to the TRIPS (Trade Related Aspects of Intellectual Property Rights) agreement which is applicable to the WTO (World Trade Organization) participating countries other labels such as Certification Mark and Collective Mark are also available for non-EU countries. Within this context and considering the special features that characterize local landraces and the cultural heritage and connection with specific regions, it is of pivotal importance to further investigate and point out these features that could result in high added value products and the reinforcement of rural economies.

Therefore, the prospect of valorization of landraces and traditional crop varieties seems very promising toward a sustainable and quality-orientated horticulture.

This e-book aims to highlight the significance of conserving local landraces toward shaping future horticultural production in a sustainable framework and point out all these special features that characterize local landraces and make them valuable, especially under harsh conditions and/or abiotic and biotic stress factors. In this context, Major et al.  tried to differentiate 13 shallot landraces preserved along the Croatian coast by using morphological characters, while the authors also studied the chemical composition of the tested landraces. After using a multivariate classification, it was suggested that Croatian shallot landraces can be classified into three distinct groups (*Allium cepa* Aggregatum, *A*. x *proliferum*, and *A*. x *cornutum*), while biochemical profiles and morphological parameters could be used for species identification. “Calçots” are a typical Spanish product which is produced by immature floral stems of onion landrace “Blanca Tardana de Lleida” resprouts. In the study of Sans et al., the effect of pre-harvest factors (genotype and growing conditions) on chemical composition and sensorial properties of calçots was evaluated by assessing the original genotype and three new genotypes derived from the original one under different conditions. Based on the results, modulation of cultivation practices, growing conditions, and breeding selection may increase yield and quality of the final product. Another landrace that is described in this e-book is “Carota di Polignano,” a multi-colored Italian landrace of carrot which was evaluated for the possibility of biofortifying with iodine by Signore et al. under open field and greenhouse conditions. The results of that study demonstrated that open field cultivation allows for better iodine enrichment of the final product, thus Recommended Daily Allowance may be achieved easier through the consumption of lower amounts of these carrots.

Tomato is the most important fruit vegetable and many local landraces are preserved throughout the world as local or farmer varieties. In their study, Massaretto et al. evaluated the potential of using two tomato landraces (Negro Yeste and Verdal), endemic of the Spanish Southeast area which is characterized by harsh climatic conditions, as alternatives to commercial varieties for cultivation under saline growing conditions. High adaptability of local landraces to salinity was observed, since both landraces exhibited high fruit quality and yield comparing to the commercial cultivar tested under the same conditions. Similarly, Figás et al. evaluated 12 long shelf-life tomato genotypes, including seven landraces, for suitability under open field and protected environment growing conditions. The variation in chemical composition, plant morphology, and agronomic performance revealed that growing conditions have a high impact on shelf-life of tomato fruit. In that way, landraces could be a useful material in breeding programs focusing on adaptation of tomato genotypes to greenhouse cultivation.

On the other hand, the interest in leafy vegetable landraces is also discussed in the present e-book.  Casals Missio et al. evaluated agronomic and quality features as well genetic variability of 32 lettuce genotypes, including landraces and modern varieties, in organic farming agrosystems. Moreover, farmers and consumers participated in genotype characterization in terms of yield, susceptibility to *Bremia lactucae* infections, visual appearance, and taste. According to the results, modern lettuce varieties and landraces were clearly distinguished, mostly due to significant differences in marketable weight and tolerance to infestations by *B. lactucae*, while farmers and consumers showed high capacity for phenotype characterization and they should be included in future research projects for local landraces recovery. Moreover, Renna et al. performed studies of nutritional value, mineral composition and antioxidant activity evaluation, as well as ethnobotanical surveys of various unconventional vegetables traditionally used in Puglia (Southern Italy). The studied vegetables included offshoots of two globe artichoke landraces, greens of summer squash and faba beans landraces, and crenate broomrape. Two more research articles and two mini reviews highlighted the importance of genetic diversity of legume germplasm. In particular, Cullis et al.  presented the features that allow marama bean, an orphan legume indigenous to Southern Africa (Kalahari Desert), to survive under harsh conditions. It was also suggested the importance of unveiling the acclimatization mechanisms to improve the performance of major crops under a climate change regime. The next mini review authored by De Ron et al. addressed the importance of conservation of common bean, runner bean, and cowpea germplasms for the genetic improvement of varieties toward the sustainable production of legumes. The recovery of “Caparrona” or “Caparrona de Monzón” common bean landrace for commercial purposes concerned Mallor et al. who performed both *in situ* and *ex situ* experiments after collection of seed samples of the aforementioned landrace. From all the collected seed samples, only two of them were finally used to register and commercialize “Caparrona” beans as a gourmet product by a local producers' association. In the next original research article authored by Rivera et al., a Spanish core collection of 202 common bean landraces was evaluated in terms of chemical composition, showing a high genetic diversity. The results also showed no significant correlation between chemical composition and sensorial quality which could be further valorized in the development of elite cultivars with superior nutritional composition and sensory traits. Finally, Marconi et al. performed a genetic characterization of 175 apple accessions from Central Italy, including local, modern, and ancient varieties, by using 19 Single Sequence Repeats (SSR) markers. The results showed a significant genetic variation among the tested accessions, while duplicates, synonyms, and homonyms were also identified.

Modern horticulture faces serious challenges due to climate change and the increasing market demands for food quality and food safety. Local landraces could be proven a useful tool toward shaping a sustainable and quality-oriented horticulture. We believe that the present e-book will raise the scientific interest to local landraces of horticultural species in order to reveal their importance to crop adaptation to the ongoing climate change, as well as their socio-economic impact on rural communities. We also believe that future research concerning local landraces of horticultural species is of pivotal importance in order to unveil their special features, to evaluate these genotypes under intensified cultivation systems and to include them in breeding programs for the production of new elite genotypes.

## Author Contributions

All authors listed have made a substantial, direct and intellectual contribution to the work, and approved it for publication.

### Conflict of Interest Statement

The authors declare that the research was conducted in the absence of any commercial or financial relationships that could be construed as a potential conflict of interest.

